# Human Trifunctional Protein Alpha Links Cardiolipin Remodeling to Beta-Oxidation

**DOI:** 10.1371/journal.pone.0048628

**Published:** 2012-11-09

**Authors:** William A. Taylor, Edgard M. Mejia, Ryan W. Mitchell, Patrick C. Choy, Genevieve C. Sparagna, Grant M. Hatch

**Affiliations:** 1 Department of Pharmacology and Therapeutics, Center for Research and Treatment of Atherosclerosis, Manitoba Institute of Child Health, University of Manitoba, Winnipeg, Manitoba, Canada; 2 Biochemistry and Medical Genetics, Center for Research and Treatment of Atherosclerosis, Manitoba Institute of Child Health, University of Manitoba, Winnipeg, Manitoba, Canada; 3 Department of Integrative Physiology, University of Colorado at Boulder, Boulder, Colorado, United States of America; Simon Fraser University, Canada

## Abstract

Cardiolipin (CL) is a mitochondrial membrane phospholipid which plays a key role in apoptosis and supports mitochondrial respiratory chain complexes involved in the generation of ATP. In order to facilitate its role CL must be remodeled with appropriate fatty acids. We previously identified a human monolysocardiolipin acyltransferase activity which remodels CL via acylation of monolysocardiolipin (MLCL) to CL and was identical to the alpha subunit of trifunctional protein (αTFP) lacking the first 227 amino acids. Full length αTFP is an enzyme that plays a prominent role in mitochondrial β-oxidation, and in this study we assessed the role, if any, which this metabolic enzyme plays in the remodeling of CL. Purified human recombinant αTFP exhibited acyl-CoA acyltransferase activity in the acylation of MLCL to CL with linoleoyl-CoA, oleoyl-CoA and palmitoyl-CoA as substrates. Expression of αTFP increased radioactive linoleate or oleate or palmitate incorporation into CL in HeLa cells. Expression of αTFP in Barth Syndrome lymphoblasts, which exhibit reduced tetralinoleoyl-CL, elevated linoleoyl-CoA acylation of MLCL to CL *in vitro*, increased mitochondrial respiratory Complex proteins and increased linoleate-containing species of CL. Knock down of αTFP in Barth Syndrome lymphoblasts resulted in greater accumulation of MLCL than those with normal αTFP levels. The results clearly indicate that the human αTFP exhibits MLCL acyltransferase activity for the resynthesis of CL from MLCL and directly links an enzyme of mitochondrial β-oxidation to CL remodeling.

## Introduction

Trifunctional protein is a multifunctional, membrane-bound enzyme protein catalyzing three enzyme activities - long-chain enoyl-Coenzyme A hydratase, long-chain 3-hydroxyacyl-Coenzyme A-dehydrogenase and long-chain 3-oxoacyl-Coenzyme A thiolase [1], [2]. Trifunctional protein plays a key role in beta-oxidation of long chain fatty acids for production of energy in the form of ATP in mitochondria.

Cardiolipin (CL) is a major phospholipid found in mammalian mitochondria and is important for the modulation of the activity of several mitochondrial enzymes involved in the generation of ATP [3]. CL has been implicated in the intrinsic pathway of apoptosis [4] and is required for caspase-8 cleavage of Bid at the mitochondrial outer membrane [5]. We recently showed that stomatin like protein-2, a widely expressed mitochondrial inner membrane protein of previously unknown function, expression in T lymphocytes resulted in increased resistance to apoptosis through the intrinsic pathway [6]. Alteration in content of CL has been shown to alter oxygen consumption in mitochondria [7], [8]. For example, in rat heart subjected to ischemia and reperfusion the reduction in electron transport chain activity was coupled with a reduction in CL [9]. Under experimental conditions in which CL is removed or digested away from mitochondrial respiratory chain proteins by phospholipases, denaturation and complete loss in activity occurred [10]. The prohibitins are an evolutionarily conserved and ubiquitously expressed family of membrane proteins that are essential for cell proliferation and development in higher eukaryotes [11], [12]. Prohibitin complexes function as protein and lipid scaffolds that ensure the integrity and functionality of the mitochondrial inner membrane and they associate with CL. CL is important for formation of the prohibitin-m-AAA protease complex, the alpha-ketoglutarate dehydrogenase complex and mitochondrial respiratory chain supercomplexes [13]. Stomatin like protein-2 interacts with prohibitin-1 and -2 and binds to CL to facilitate formation of metabolically active mitochondrial membranes [6]. The fatty acyl molecular composition of CL appears to be important for the biological function of CL [10], [14]. The major tetra-acyl molecular species found in rat liver (approximately 57% of total) and bovine heart (approximately 48% of total) are (18∶2-18∶2)-(18∶2-18∶2) whereas in human heart this may be as high as 80% [15]. Remodeling of CL is essential to obtain this enrichment of CL with linoleate since the enzymes of the CL biosynthetic pathway exhibit little molecular species substrate specificity [16], [17]. Alterations in the molecular composition of CL are associated with various disease states including diabetes and Barth Syndrome (BTHS) [18], [19]. BTHS is a rare X-linked genetic disorder associated with cardiomyopathy, cyclic neutropenia, 3-methylglucaconic aciduria and mild hypocholesterolemia and is the only disease in which the specific biochemical defect is a reduction in CL [20], [21]. The decrease in CL is caused by a mutation in the BTHS gene TAZ. TAZ codes for the protein tafazzin. Tafazzin remodels newly synthesized CL with linoleic acid. In patients with BTHS the ability to remodel CL is reduced. In summary, maintenance of the appropriate content and fatty acyl composition of CL in mitochondria is essential for proper cellular function.

**Figure 1:**
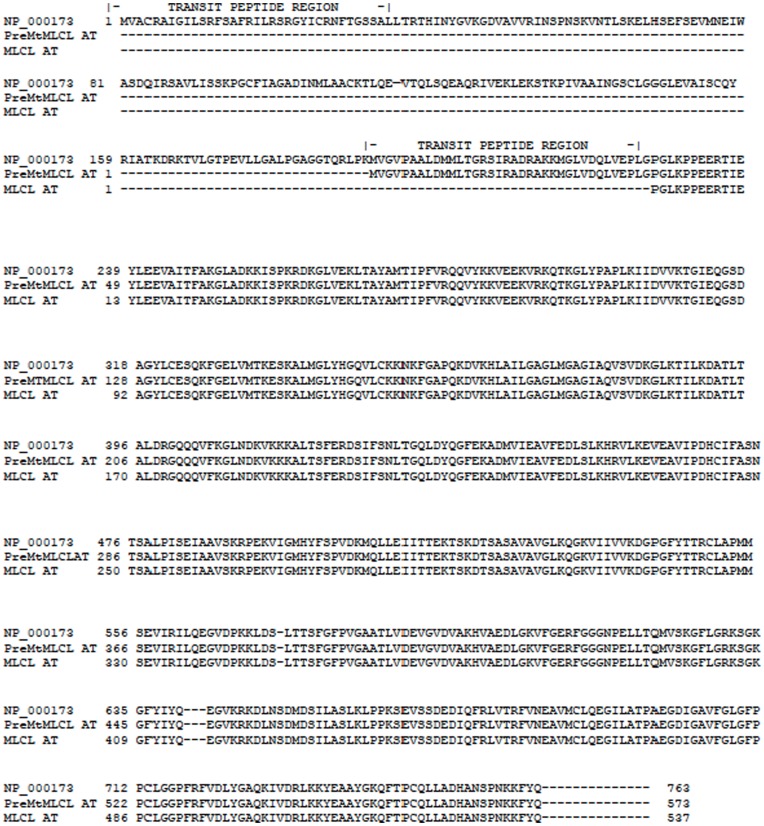
Alignment of human αTFP (NP_000173) with MLCL AT-1.

Currently, there are only three known enzymes that directly remodel CL 1. *Monolysocardiolipin acyltransferase-*1 (MLCL AT-1), which we identified as an unknown 59 kDa human protein (AXX93141.1) exhibiting structural identity to the human alpha subunit of trifunctional protein (αTFP) lacking the first N-terminal 227 amino acids [22] (**Figure 1**), 2. *Tafazzin*, a mitochondrial CL transacylase reaction first described in rat liver [23] that has been found to be the defective gene product in Barth Syndrome [24], and 3. *acyllysocardiolipin acyltransferase-1* (*ALCAT1)* [25]. ALCAT-1 may play a role in the early specification of hematopoietic and endothelial cells [26], [27] through acyl-Coenzyme A-dependent reacylation of MLCL to CL in microsomes [25]. Here we characterize a possible *fourth* CL remodeling enzyme, mitochondrial αTFP, which also exhibits *in vitro* and *in vivo* MLCL AT activity. With the findings in this study, we provide a link between mitochondrial β-oxidation and CL remodeling.

## Experimental

### Materials and Methods

[1-^14^C]Linoleic acid, [1-^14^C]oleic acid, [^3^H]palmitic acid, [1-^14^C]linoleoyl-Coenzyme A, [1-^14^C]oleoyl-Coenzyme A and [1-^14^C]palmitoyl-Coenzyme A were obtained from either Dupont, Mississauga, Ontario, or Amersham, Oakville, Ontario, Canada or American Radiolabeled Chemicals Inc., St. Louis, MO. DMEM and fetal bovine serum were products of Canadian Life Technologies (GIBCO), Burlington, Ontario, Canada. Lipid standards were obtained from Serdary Research Laboratories, Englewood Cliffs, New Jersey, USA. Monolysocardiolipin (MLCL) was obtained from Avanti Polar Lipids, Alabaster, NY., USA. Thin layer chromatographic plates (silica gel G, 0.25 mm thickness) were obtained from Fisher Scientific, Winnipeg, Canada. Ecolite scintillation cocktail was obtained from ICN Biochemicals, Montreal, Quebec, Canada. HeLa cells were obtained from American Type Culture Collection. Epstein-Barr virus transformed age-matched control or BTHS lymphoblasts were obtained from the Coriell Institute for Medical Research, Camden, New Jersey. Anti-αTFP antibody was a generous gift from Dr. Zaza Khuchua, Children’s Hospital Medical Center, Cincinnati, OH. MitoProfile® Total OXPHOS antibody cocktail was obtained from Abcam Inc., Toronto, Ontario. Western blotting analysis system was used for protein expression studies and was obtained from Amersham Pharmacia Biotech UK Limited, Buckinghamshire, England. Kodak X-OMAT film was obtained from Eastman Kodak Co., Rochester, NY., USA. QIAGEN OneStep RT-PCR kit was used for PCR studies. All other chemicals were certified ACS grade or better and obtained from Sigma Chemical Company, St. Louis, USA or Fisher Scientific, Winnipeg, Manitoba, Canada.

#### Preparation and purification of recombinant αTFP for western blot analysis and in vitro enzyme assays

The full length primers for the Homo sapiens hydroxyacyl coenzyme A dehydrogenase αTFP (NP_000173) subunit containing a 6× HIS-tag in the reverse primer without stop codon was prepared from Invitrogen (custom primer design) (**Table 1**). The HIS-tag was required for binding of the protein to the Ni-NTA affinity resin (see below). The primers were amplified using 1 ug HeLa cell RNA. The cDNA’s containing the full length sequences were inserted into pcDNA 3.1 using the TOPO-Cloning Reaction with pEXP5-CT/TOPO vector (Invitrogen). *E. coli* ONE SHOT bacteria (Invitrogen) were transformed with the construct chemically with S.O.C. medium (Invitrogen), and inoculated onto ampicillin containing agar for growth overnight. In the morning, the colonies were inoculated into 5 ml of ampicillin containing L-B medium and cultured at 37°C in an orbital shaker at 220 rpm overnight. The plasmid was purified from the *E. coli* using The MidiPrep Kit (Invitrogen). The sequence of the plasmids were verified by PCR using the specific primers and also by a DNA sequencer (Manitoba Institute of Cell Biology). The recombinant protein was expressed using the Cell-free *E. coli* Expression System (Invitrogen). The recombinant protein was purified with a Ni-NTA affinity resin (Fisher). The resin was first pre-eluted with high salt (1 M NaCl) and low imidazole concentration (20 mM) and then the protein eluted with 200 mM imidazole. The protein eluate from the Ni-NTA affinity resin was then further purified by MLCL-adriamycin agarose affinity chromatography and eluted from the column with MLCL as previously described [22].

**Table 1:** Primers used for human recombinant αTFP protein synthesis, plasmid transfections, realtime PCR and RNAi sequences.

Primers for recombinant protein synthesis:
αTFP:
Forward, (bp 237–256) CGG AAC AAG GGA TGA CCA GAA CCC ATA TTA AC
Reverse, (bp 2419–2390) TGA GTC AAG GGG TAG AAC TTC TTG TTA GGG CT
Plasmid primers for transfection experiments:
αTFP (Nucleotide accession no. NM_000182):
Forward, (bp 131–160) ATG ACC AGA ACC CAT ATT AAC
Reverse, (bp 2419–2390) CTG GTA GAA CTT CTT GTT AGG GCT GTT AGC
Primers used for real time PCR:
Human
αTFP
Forward, TCG GCA TCT GGG TTT TAG TC
Reverse, GGT CAG CAA AGC AGA AGA CC
18s ribosomal RNA
Forward, GCA ATT ATT CCC CAT GAA CG
Reverse, GGC CTC ACT AAA CCA TCC AA
Rat
αTFP
Forward, GCC GTC CTT ATT TCG TCA AA
Reverse, GCT ATG GCA AGC TCA AGT CC
18s ribosomal RNA
Forward, CAT TCG AAC GTC TGC CCT AT
Reverse, GCC TTC CTT GGA TGT GGT AG
RNAi sequences:
Mock: TTG ACT CCA TAG TTA ATA TGG GTT C
RNAi1: GAA CCC ATA TTA ACT ATG GAG TCA A
RNAi2: TGT TCG AAT TAA CTC TCC CAA TTC A

#### Culture, radiolabeling and harvesting of HeLa cells and barth syndrome lymphoblasts

HeLa cells were grown in DMEM containing 10% fetal bovine serum. To evaluate the effect of αTFP on CL acylation with different species of radioactive fatty acid, 13 µg of αTFP protein plasmid in 23 µl of Lipofectamine (Invitrogen) were added to HeLa cells at 50% confluence. Plasmid primers used for αTFP transfection are shown in **Table 1**. After 24 h, 0.1 mM (1 µCi/dish) of fatty acid ([1-^14^C]linoleic acid or [1-^14^C]oleic acid or [^3^H]palmitate (bound to bovine serum albumin in a 1∶1 molar ratio) was added and incubation continued for another 24 h. Cells were then harvested and radioactivity incorporated into CL determined as described [22]. In other experiments, cells were washed twice with ice cold saline and harvested with 2 ml lysis buffer (10 mM Tris-HCL, pH 7.4, 0.25 M sucrose) and then homogenized with 30 strokes of a Dounce A homogenizer. The homogenate was centrifuged at 1,000 *g* for 5 min and the supernatant centrifuged at 10,000 *g* for 15 min. The pellet was resuspended in 0.5 ml homogenization buffer and used for assay of MLCL AT activity as described below.

Age-matched control and BTHS lymphoblasts were grown in suspension in RPMI-1640 medium containing 10% fetal bovine serum until reaching a concentration of 10^6^ cells/ml. Lymphoblasts were pelleted and placed in Opti-MEM (Invitrogen) (5×10^6^ cells/ml) and incubated with 40 µg of αTFP or MLCL AT-1 plasmid and electroporation was performed at 950 µFarads, 250 volts, for 23 msec in 800 µl of Opti-MEM using a BTX Electroporation System Electrocell Manipulator 600. Plasmid primers used for αTFP are shown in **Table 1** and primers for MLCL AT-1 were previously described [22]. Cells were then incubated for 24 h with 0.1 mM linoleic acid bound to albumin (1∶1 molar ratio) and total RNA isolated and αTFP mRNA expression determined using real time-PCR (Eppendorff realplex^2^) and were expressed relative to 18s ribosomal RNA. Human primers for real time-PCR are shown in **Table 1**. In other experiments, fatty acyl molecular species of CL in normal and BTHS lymphoblasts was quantified using electrospray ionization mass spectrometry coupled to HPLC as previously described [28].

In other experiments, BTHS lymphoblasts were transfected with two different αTFP RNAi nucleotide sequences generated from upstream nucleotide regions 243–267 (RNAi1) and 283–307 (RNAi2) and CL and MLCL levels quantified using electrospray ionization mass spectrometry as above. The ON- and OFF-target RNAi to human αTFP was prepared from Invitrogen using BLOCK-iT RNAi Designer program. The off-target sequence for mock-transfection (Mock) and the on-target sequences (RNAi1, RNAi2) for RNAi transfection used are shown in **Table 1**. In other experiments, the αTFP RNAi sequences were transfected into HeLa cells as previously described [22] and mRNA expression of αTFP or MLCL AT-1 determined using either semi-quantitative or real-time PCR.

#### Determination of enzyme activities

MLCL AT activity was determined as described previously [29], [30]. Essentially, human recombinant αTFP (20 ng) or HeLa cell or age-matched control and BTHS lymphoblast mitochondrial protein (20 µg) was incubated in 50 mM TRIS-HCL buffer pH 8.0 and incubated with 0.3 mM MLCL and [1-^14^C]linoleoyl-Coenzyme A (120,000 dpm/nmol) at 37°C for 1 h or longer for smaller protein quantities. The reaction was stopped by the addition of chloroform:methanol (2∶1). The organic fraction was isolated by centrifuging the mixture after the addition of 0.9% KCL. After an additional washing of the organic fraction with theoretical upper phase, the chloroform layer was dried with nitrogen, resuspended in 25 µl chloroform:methanol (2∶1) and applied to a Whatman silica gel coated glass thin layer plate with CL standard. [^14^C]CL was isolated by 2-dimensional chromatography using the following solvent mixtures: first dimension (chloroform:methanol:water, 65∶25:4, by vol) and second dimension (chloroform:acetone:methanol:acetic acid:water, 50∶20:10∶10:5, by vol). CL was visualized with iodine vapor and silica gel corresponding to the CL spot removed and placed into scintillation vials containing 5 ml Ecolite scintillation cocktail and radioactivity determined in a LS 6500 Liquid Scintillation counter (Beckman). In some experiments, MLCL AT activity was determined with various lysophospholipids or MLCL and varying concentrations of [1-^14^C]linoleoyl-Coenzyme A or [1-^14^C]oleoyl-Coenzyme A or [1-^14^C]palmitoyl-Coenzyme A in the presence of human recombinant αTFP.

#### Electrophoresis and western blot analysis

Purified human recombinant αTFP was separated on the Bio Rad mini gel electrophoresis system by SDS-PAGE (10% acrylamide) as previously described [30]. The electrophoresis was performed using synthetic pre-stained molecular markers from BioRad. After the electrophoresis, the proteins were transferred onto PVDF membranes, using TRIS-glycine buffer (pH 8.3) with 20% methanol, at 15 V for 1.5 h. The proteins were probed overnight with polyclonal pig liver anti-MLCL AT antibody [22], [30]. The second antibody was anti-rabbit IgG. The protein was visualized on X-OMAT film by chemiluminescence (Amersham). In some experiments, HeLa cells were mock transfected or transfected with αTFP and Western blot analysis performed using anti- αTFP antibody as described above with β-actin used as a loading control. In other experiments, BTHS lymphoblasts were mock transfected or transfected with recombinant αTFP and mitochondrial fractions prepared and αTFP protein expression determined using the anti-αTFP antibody or mitochondrial Complex I-V protein subunit levels determined using the MitoProfile® Total OXPHOS antibody cocktail according to the manufacturer’s instructions.

#### Preparation of hyper- and hypothyroid animals

Male Sprague Dawley rats (125–175 g) were used and were housed in a temperature and light controlled room. They were maintained on Purina rat chow and tap water *ad libitum*. All animals were kept in identical housing units on a cycle of 12 h of light and 12 h of darkness. Rats were made hypothyroid by administration of (0.5% w/v) 6-n-propyl-2-thiouracil (PTU) in their drinking water for 34 days. Rats were made hyperthyroid by i.p. administration of 250 µg/Kg/day thyroxine (T_4_) for 5 days. Livers were isolated from euthyroid, hyper- and hypothyroid animals and total RNA prepared and αTFP mRNA expression determined under the conditions described [22]. αTFP mRNA expression were expressed relative to 18s ribosomal RNA. The nucleotide sequences for the rat αTFP and 18S primers are shown in **Table 1**. In some experiments, rat liver mitochondrial fractions from euthyroid and hyperthyroid animals were prepared and subjected to Western blot analysis using the anti-αTFP antibody as described above.

#### Ethics statement

Treatment of animals conformed to the Guidelines of the Canadian Council on Animal Care. This study was approved by the Bannatyne Campus Protocol Management and Review Committee of the University of Manitoba.

#### Other determinations

Protein was determined as described [31]. Student’s t-test was used for determination of statistical significance. The level of significance was defined as p<0.05 unless otherwise indicated.

## Results

The alpha subunit of trifunctional protein exhibits acyl-Coenzyme A-dependent MLCL AT enzyme activity and is a MLCL acyltransferase specific for CL remodeling in vivo in HeLa cells.

**Figure 2:**
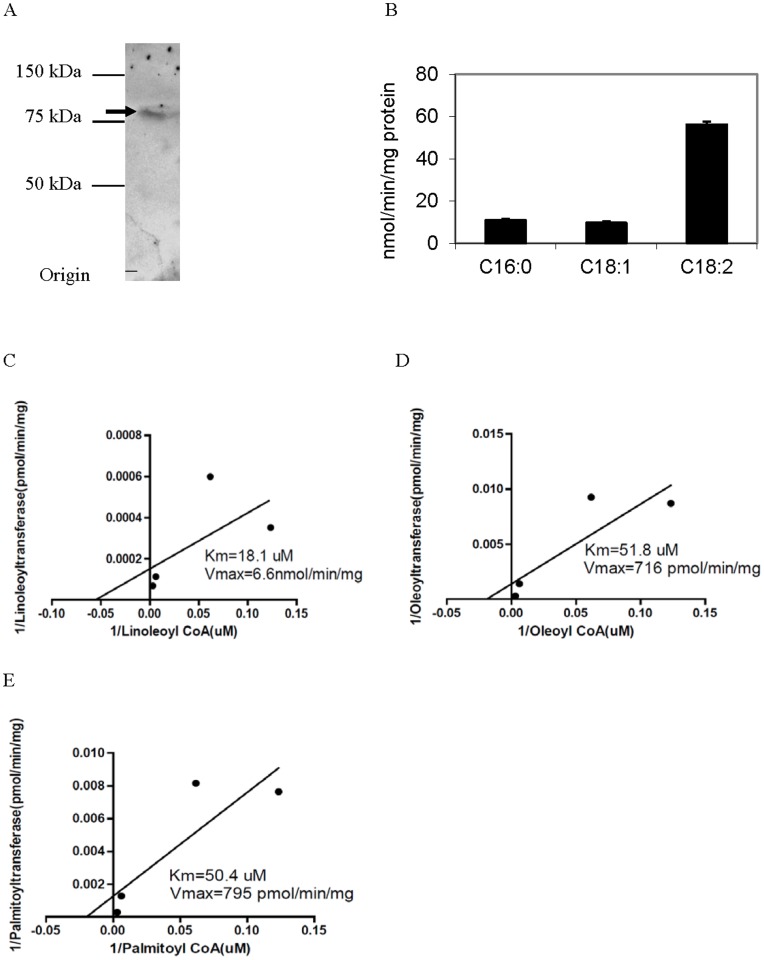
Western blot analysis of the HIS-tagged human recombinant αTFP and MLCL AT activity of the HIS-tagged human recombinant αTFP in presence of palmitoyl-Coenzyme A or oleoyl-Coenzyme A or linoleoyl-Coenzme A. A. HIS-tagged human recombinant αTFP was generated, purified and 2 µg protein subjected to SDS-PAGE and then western blot analysis performed using the polyclonal antibody to MLCL AT-1 as described in Materials and Methods. A representative blot is shown. Molecular mass markers are indicated on the left. Arrow indicates the HIS-tagged recombinant αTFP. B. MLCL AT *in vitro* activity of purified recombinant αTFP was determined in presence of [1-^14^C]palmitoyl-Coenzyme A (C16∶0) or [1-^14^C]oleoyl-Coenzyme A (C18∶1) or [1-^14^C]linoleoyl-Coenzme A (C18∶2) as described in Materials and Methods. Data represent the mean ± standard deviation of three experiments. HIS-tagged human recombinant αTFP was generated, purified and MLCL AT *in vitro* activity was determined in the presence of MLCL and various concentrations of linoleoyl-Coezyme A (C) or oleoyl-Coenzyme A (D) or palmitoyl-Coenzyme A (E) as described in Materials and Methods. Data represent the mean of two experiments assayed in duplicate.

Previously we identified and characterized an acyl-Coenzyme A-dependent MLCL AT (MLCL AT-1) [22]. This 59 kDa protein was identical to the 74 kDa αTFP *minus the first 227 amino acids*. Alignment of the full length human αTFP protein with MLCL AT-1 revealed the presence of mitochondrial transit peptides in both αTFP and MLCL AT-1 (**Figure 1**). In the current study, we initially examined the protein characteristics of αTFP. Human recombinant HIS-tagged αTFP was generated, purified using the HIS-tag and then western blot analysis was performed using the polyclonal antibody to MLCL AT-1 [22]. Western blot analysis indicated the presence of the 79 kDa HIS-tagged αTFP recombinant (**Figure 2A**). Thus, the polyclonal antibody to the MLCL AT-1 cross-reacted with αTFP.

Recombinant protein for human αTFP was prepared, purified and examined for MLCL AT activity using MLCL with [1-^14^C]oleoyl-Coenzyme A or [1-^14^C]linoleoyl-Coenzyme A or [1-^14^C]palmitoyl-Coenzyme A as substrates. αTFP exhibited MLCL AT activity with all substrates but activity was highest with [1-^14^C]linoleoyl-Coenzyme A (**Figure 2B**). Thus, αTFP exhibits MLCL AT activity with various acyl-Coenzyme A substrates but appeared to have a greatest activity for linoleoyl-Coenzyme A as substrate *in vitro*.

The lysophospholipid and Coenzyme A specificity and kinetics of αTFP MLCL AT activity were then determined. There was no detectable acyltransferase activity in the presence of lysophosphatidylcholine, lysophosphatidylethanolamine, lysophosphatidylglycerol or lysophosphatidic acid (**Table 2**). Thus, αTFP MLCL AT activity exhibits specificity for MLCL. αTFP MLCL AT activity was then determined in the presence of MLCL and various concentrations of linoleoyl-Coenzyme A or oleoyl-Coenzyme A or palmitoyl-Coenzyme A. The Km of the enzyme was 18.1 µM, 51.8 µM and 50.4 µM when assayed with linoleoyl-Coenzyme A or oleoyl-Coenzyme A or palmitoyl-Coenzyme A as substrates, respectively. (**Figure 2C, 2D, 2E**). The Vmax of the enzyme was 6,600, 716 and 795 pmol/min/mg protein when assayed with linoleoyl-Coenzyme A or oleoyl-Coenzyme A or palmitoyl-Coenzyme A as substrates, respectively. Thus, αTFP MLCL AT activity exhibited higher specificity for linoleoyl-Coenzyme A *in vitro*.

**Table 2:** Lysophospholipid specificity of αTFP.

Lysophospholipid	Enzyme activity (pmol/min/mg protein)
Monolysocardiolipin	56.3±1.3
Lysophosphatidic acid	ND
Lysophosphatidylglycerol	ND
Lysophosphatidylcholine	ND
Lysophosphatidylethanolamine	ND

Human recombinant αTFP linoleoyl-CoA acyltransferase activity was determined in the presence of various lysophospholipids as described in Materials and Methods. ND, not detected. Values represent the mean ± standard deviation of three experiments.

**Figure 3:**
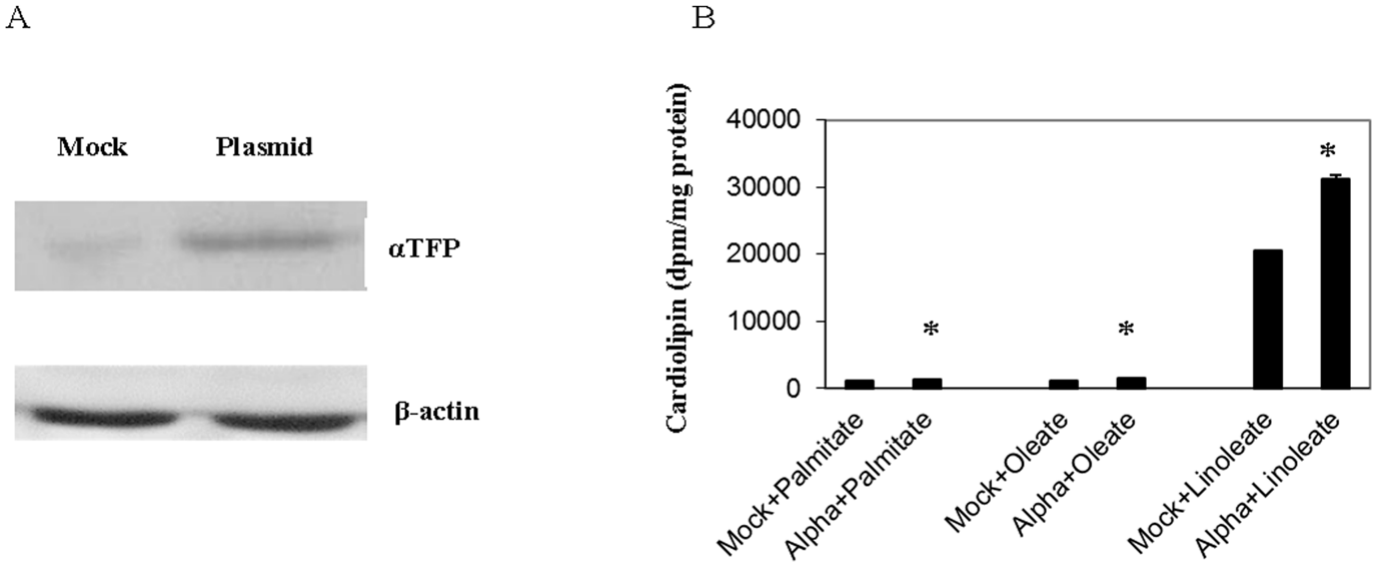
Western blot analysis of αTFP and incorporation of fatty acids into CL in HeLa cells transfected with human recombinant αTFP. HeLa cells (A) were mock transfected (Mock) or transfected with human recombinant αTFP plasmid (Plasmid) and mitochondrial fractions prepared and 25 µg protein subjected to SDS-PAGE followed by western blot analysis using anti-αTFP antibody or anti-β-actin antibody as described in Materials and Methods. A representative blot is depicted. Hela cells (B) were mock transfected (mock) or transfected with human recombinant αTFP (alpha) and incubated with for 4 h with [1-^14^C]linoleate (linoleate) or [1-^14^C]oleate (oleate) or [^3^H]palmitate (palmitate) and radioactivity incorporated into CL determined. Data represent the mean ± standard deviation of three experiments. *p<0.05.

To determine if αTFP promoted fatty acid incorporation into CL *in vivo*, HeLa cells were transfected with recombinant αTFP and then incubated for 24 h with [1-^14^C]linoleate or [1-^14^C]oleate or [1-^14^C]palmitate and radioactivity incorporated into CL determined. Western blot analysis with antibody to αTFP indicated that transfection of HeLa cells with αTFP resulted in increased expression of the αTFP protein compared to control (**Figure 3A**). Densitometry analysis indicated an approximate 2.2-fold increase in αTFP protein (data not shown). Transfection of HeLa cells with αTFP resulted in a significant 56% increase (p<0.05) in MLCL AT *in vitro* activity from 37±5 nmol/min/mg protein to 58±7 nmol/min/mg protein (n = 4). Expression of αTFP in HeLa cells significantly increased [1-^14^C]linoleate incorporation into CL by 52% compared to control (p<0.013) (**Figure 3B**). In addition, expression of αTFP in HeLa cells increased [1-^14^C]oleate (1232±46 mock vs 1492±132 αTFP dpm/mg protein, p<0.043) and [^3^H]palmitate (1073±79 mock vs 1241±48 αTFP dpm/mg protein, p<0.048) incorporation into CL. Thus, αTFP promotes fatty acid incorporation into CL with highest specificity for linoleate *in vivo*.

Expression of αTFP in BTHS lymphoblasts increases MLCL AT activity, mitochondrial respiratory chain protein subunits and elevates unsaturated fatty acyl molecular species of CL.

**Figure 4:**
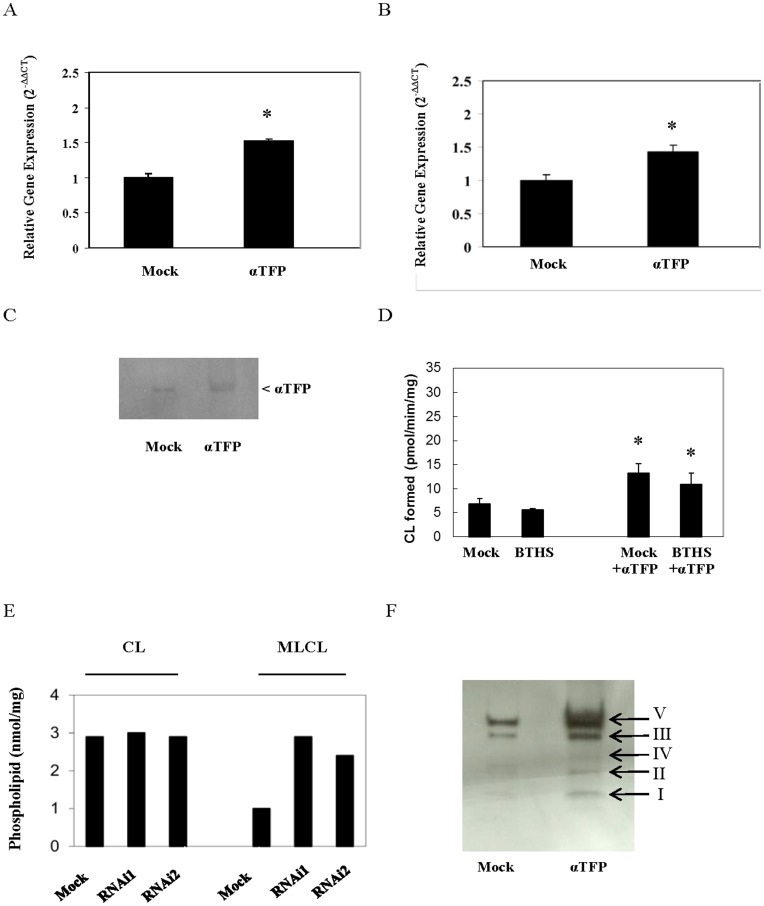
mRNA and protein expression of αTFP in control and/or BTHS lymphoblasts transfected with αTFP, MLCL AT activity in control and BTHS lymphoblasts transfected with αTFP, MLCL levels in BTHS lymphoblasts transfected with αTFP RNAi and mitochondrial respiratory chain subunits in BTHS lymphoblasts transfected with αTFP. Age-matched control lymphoblasts (A) or BTHS lymphoblasts (B) were mock transfected (Mock) or transfected with αTFP plasmid (αTFP) and mRNA expression of αTFP determined as described in Materials and Methods. Data represents the mean ± standard deviation of four experiments. *p<0.05. C. BTHS lymphoblasts were mock transfected (Mock) or transfected with human recombinant αTFP plasmid (αTFP) and mitochondrial fractions prepared and 150 µg protein subjected to SDS-PAGE followed by western blot analysis using the anti-αTFP antibody as described in Materials and Methods. A representative blot is depicted. D. Control (Mock) and BTHS (BTHS) lymphoblasts were mock transfected or transfected with αTFP plasmid (+αTFP) and MLCL AT activity determined. Data represent the mean ± standard deviation of three experiments. *p<0.05. E. BTHS lymphoblasts were mock transfected (Mock) with off-target αTFP nucleotide sequence or transfected with two different on-target αTFP RNAi nucleotide sequences (RNAi1, RNAi2) and CL and MLCL levels determined. Data represents the mean of two experiments. F. BTHS lymphoblasts were mock transfected (Mock) or transfected with human recombinant αTFP (αTFP) and mitochondrial fractions prepared and 40 µg protein subjected to SDS-PAGE followed by western blot analysis for respiratory chain protein subunits as described in Materials and Methods.

BTHS cells exhibit a reduction in L_4_-CL levels due to a mutation in *TAZ*, the CL transacylase which remodels CL with linoleate [19]–[21]. However, BTHS cells are not completely devoid of CL. We previously showed that expression of MLCL AT-1 elevated total CL levels in BTHS lymphoblasts [22]. However, in that study we did not examine if expression of MLCL AT-1 in BTHS lymphoblasts increased linoleate species of CL. We thus examined if expression of αTFP or MLCL AT-1 in aged-matched control and BTHS lymphoblasts would increase linoleate-containing species of CL. Initially we examined if transfection of BTHS lymphoblasts with αTFP increased αTFP mRNA and protein level and MLCL AT enzyme activity. BTHS lymphoblasts or lymphoblasts from aged-matched control patients were mock transfected or transfected with human recombinant αTFP. Total RNA was isolated and αTFP mRNA expression determined using real time-PCR. In age-matched control lymphoblasts transfected with human recombinant αTFP the mRNA expression of αTFP was elevated 53% compared to mock transfected cells (**Figure 4A**). In BTHS lymphoblasts transfected with human recombinant αTFP the mRNA expression of αTFP was elevated 43% compared to mock transfected cells (**Figure 4B**). Western blot analysis revealed an increase in αTFP protein expression in BTHS lymphoblasts transfected with human recombinant αTFP (**Figure 4C**). Densitometry analysis indicated an approximate 2-fold increase in αTFP protein (data not shown). Next, mitochondrial fractions were prepared from age-matched control and BTHS lymphoblasts mock transfected or transfected with human recombinant αTFP and MLCL AT enzyme activity determined. Expression of αTFP increased MLCL AT activity in both age-matched control and BTHS lymphoblasts transfected with human recombinant αTFP compared to mock transfected cells (**Figure 4D**). We next examined if expression or knock down of αTFP would alter MLCL levels in BTHS lymphoblasts. BTHS lymphoblasts were mock transfected or transfected with human recombinant αTFP or transfected with either an off-target αTFP nucleotide sequence or two separate αTFP RNAi sequences generated from different sections of the protein and 24 h later CL and MLCL levels were determined. Expression of αTFP in BTHS lymphoblasts resulted in a modest reduction (from 1.12 to 0.81 nmol/mg protein) in MLCL compared to mock transfected cells. Knock down of αTFP in BTHS lymphoblasts did not alter CL levels but resulted in a greater accumulation of MLCL than controls (**Figure 4E**). Collectively the above studies indicate that αTFP may utilize MLCL as substrate in BTHS lymphoblasts.

Tafazzin mutations in BTHS lymphoblasts are associated with disruption and loss of mitochondrial respiratory chain complexes, particularly Complex I [32]. We examined if expression of αTFP in BTHS lymphoblasts increased mitochondrial Complex protein subunits. BTHS lymphoblasts were mock transfected or transfected with human recombinant αTFP and mitochondrial fractions isolated and mitochondrial respiratory Complex I-V protein subunit levels determined. Expression of αTFP in BTHS lymphoblasts increased expression of Complex I NDUFB8, Complex II SDH8, Complex IV-MTCO1, Complex III-UQCRC2 and Complex V-ATP5A subunits compared to mock transfected cells (**Figure 4F**). Thus, expression of αTFP in BTHS lymphoblasts elevates mitochondrial respiratory chain complex protein subunits.

**Figure 5:**
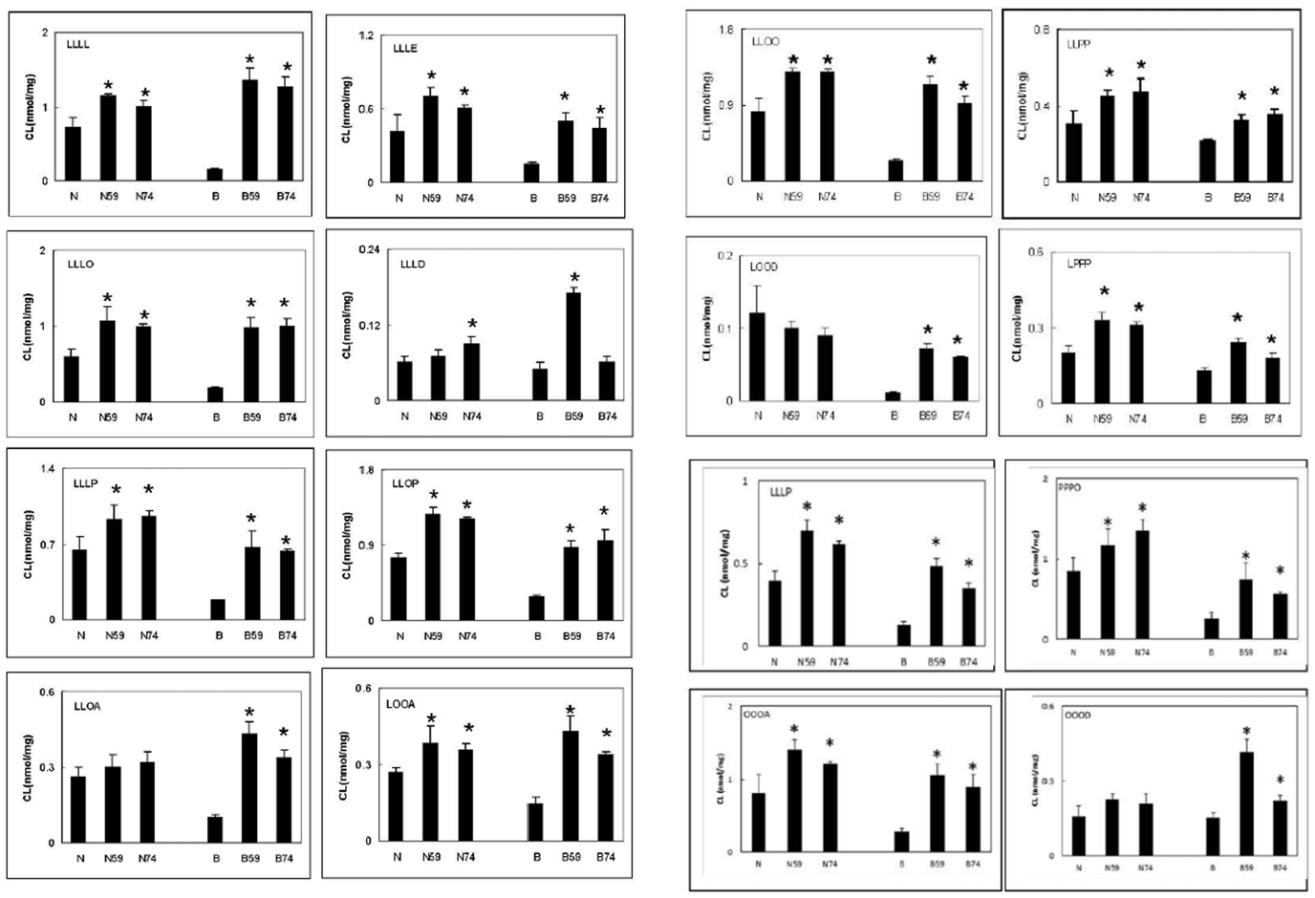
The CL fatty acyl molecular species in control and BTHS lymphoblasts expressing αTFP. Age-matched normal lymphoblasts (N) or BTHS lymphoblasts (B) were mock transfected (N, B), or transfected with the 74 kDa human recombinant αTFP plasmid (normal lymphoblasts, N74; BTHS lymphoblasts, B74) or the 59 kDa human recombinant MLCL AT-1 (normal lymphoblasts, N59; BTHS lymphoblasts, B59) and the major CL tetra-acyl molecular species determined as described in Materials and Methods. L, linoleate; O, oleate; P, palmitate; A, arachidonate; D, decosahexanoate; E, eicosapentaenoate. The tetra-acyl CL species are indicated in the upper left insert. Data represents the mean ± standard deviation of three experiments. *p<0.05.

Finally, age-matched control and BTHS lymphoblasts were mock transfected or transfected with αTFP or MLCL AT-1 and cultured for 24 h in the presence of 0.1 mM linoleate bound to albumin (1∶1 molar ratio) and the molecular species of CL determined. Expression of αTFP or MLCL AT-1 in either age-matched control or BTHS lymphoblasts resulted in an increase in unsaturated fatty acyl molecular species of CL (**Figure 5**). The above data indicate that αTFP has the ability to increase linoleate-containing species of CL in both control and BTHS lymphoblasts.

### Thyroid Hormone Regulates mRNA and Protein Expression of αTFP

**Figure 6:**
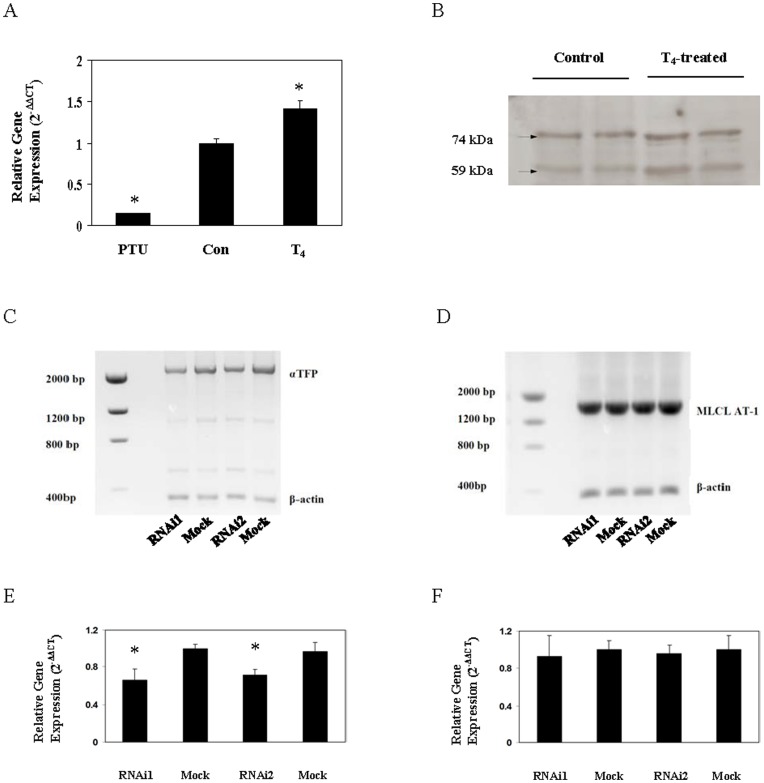
Effect of altered thyroid status on αTFP mRNA and protein expression and knock down of αTFP on αTFP and MLCL AT-1 mRNA expression. A.Rats were injected with T_4_ (250 µg/Kg) for 5 consecutive days or treated with 0.5% PTU in their drinking water for 34 days and the livers removed and total RNA prepared and αTFP mRNA expression determined as described in Materials and Methods. Con, euthyroid control; T_4_, thyroxine-treated; PTU, PTU-treated. Data represents the mean ± standard deviation of five animals. *p<0.05. B. Mitochondrial fractions from the above (Control) or T_4_-treated (T_4_-treated) animals were subjected to SDS-PAGE followed by western blot analysis using anti-αTFP antibody. A representative blot is depicted with molecular masses of the two major proteins αTFP at 74 kDa and MLCL AT-1 at 59 kDa indicated on the left. Hela cells were mock-transfected (Mock) or transfected with αTFP RNAi sequences (RNAi1, RNAi2) and αTFP mRNA expression determined by reverse transcriptase-PCR (C) or real-time PCR (E) or MLCL AT-1 mRNA expression determined by reverse transcriptase-PCR (D) or real-time PCR (F). Representative gels are shown with molecular mass markers indicated on the left in C and D with β-actin at 350 bp, MLCL AT-1 at 1,611 bp, and αTFP at 2,288 bp. In C-F the data represents the mean ± standard deviation of four experiments. *p<0.05.

Previously we observed that rat liver MLCL AT-1 expression was elevated by T_4_-treatment, which elevates plasma thyroid hormone levels, and reduced by PTU-treatment of rats, which lowers plasma thyroid hormone levels [33]. We examined if alteration in thyroid hormone status resulted in corresponding alterations in αTFP mRNA expression. Rats were injected with T_4_ (250 ug/Kg) for 5 consecutive days or treated with 0.5% PTU in their drinking water for 34 days and the livers removed and total RNA prepared and real-time PCR analysis performed. T_4_- or PTU-treatment resulted in increased or decreased heart to body weight ratio, respectively, indicating effectiveness of the protocol as we have previously described [33]. T_4_-treatment resulted in a significant increase and PTU-treatment a significant decrease in αTFP mRNA expression, respectively, compared to euthyroid controls (**Figure 6A**). Mitochondrial fractions were then prepared from either euthyroid control or T_4_-treated animals and subjected to western blot analysis using the anti-αTFP antibody. T_4_-treatment resulted in an increase in not only a 74 kDa protein band but also a prominent band at 59 kDa (**Figure 6B**). Protein sequencing analysis indicated that the 74 kDa protein was αTFP and the 59 kDa protein the previously identified MLCL AT-1. Thus, thyroid hormone modulates αTFP mRNA and protein expression.

### MLCL AT-1 is Likely Derived from αTFP

Since T_4_-treatment increased the expression of both MLCL AT-1 and αTFP as detected using the anti-αTFP antibody and the MLCL AT-1 protein sequence is identical to the αTFP protein sequence lacking the first 227 amino acids, we examined if MLCL AT-1 was related to αTFP. To characterize the relationship between αTFP and MLCL AT-1 αTFP was knocked down, using RNAi generated from upstream nucleotide regions 243–267 and 283–307 (sequences not present in MLCL AT-1), in HeLa cells and αTFP and MLCL AT-1 mRNA expression determined. RNAi knock down of αTFP in HeLa cells resulted in a significant decrease in expression of αTFP mRNA (**Figure 6C, 6E**).In contrast, RNAi knock down of αTFP in HeLa cells did not effect MLCL AT-1 mRNA expression (**Figure 6D, 6F**). The results suggest that MLCL AT-1 may possibly be a splice variant of the αTFP gene.

## Discussion

In this study we show that mitochondrial αTFP is a MLCL AT specific for the remodeling of CL. The major findings of this study are 1. The human recombinant αTFP utilized various acyl-Coenzyme A’s in the specific acylation of MLCL to CL *in vitro*, 2. Expression of the human recombinant αTFP in HeLa cells increased radioactive fatty acid incorporation into CL, 3. Expression of αTFP in BTHS lymphoblasts increased linoleoyl-CoA acylation of MLCL to CL *in vitro* and levels of mitochondrial respiratory chain protein complex subunits, 4. RNAi knock down of αTFP in BTHS lymphoblasts resulted in a further accumulation of MLCL, 5. Expression of αTFP or MLCL AT-1 in normal, or BTHS lymphoblasts which have reduced CL levels, elevated linoleate containing species of CL, and 6. MLCL AT-1 is likely derived from αTFP. The results suggest that human αTFP exhibits acyl-Coenzyme A MLCL AT activity and thus directly links a known enzyme of mitochondrial β-oxidation to CL remodeling.

The purified recombinant αTFP exhibited *in vitro* MLCL AT enzyme activity with linoleoyl-Coenzyme A, oleoyl-Coenzyme A and palmitoyl-Coenzyme A as substrates but a greater enzyme activity with linoleoyl-Coenzyme A as substrate. The Km with linoleoyl-Coenzyme A was lower than with oleoyl-Coenzyme A or palmitoyl-Coenzyme A as substates and the Vmax with linoleoyl-Coenzyme A was higher than with oleoyl-Coenzyme A or palmitoyl-Coenzyme A substrates. MLCL was the only lysophospholipid acylated by αTFP. Moreover, expression of αTFP in HeLa cells resulted in a significant increase in radioactive linoleate, oleate and palmitate incorporation into CL. However, the increase in radioactive fatty acid incorporation into CL seemed to be greatest with linoleate as substrate. These data indicate that, at least in HeLa cells, αTFP exhibits a preference for linoleoyl-Coenzyme A MLCL AT activity *in vivo*.

Previously we reported that BTHS lymphoblasts have reduced levels of CL and that expression of MLCL AT-1 in BTHS lymphoblasts elevated CL levels [22]. The underlying biochemical abnormality in BTHS is a reduction in L_4_-CL levels mediated by a mutation in the BTHS gene *TAZ* [14], [20], [21]. BTHS lymphoblasts exhibit unstable respiratory chain supercomplexes and reduced mitochondrial Complex I biogenesis [32]. Expression of human recombinant αTFP plasmid in BTHS lymphoblasts increased αTFP protein, *in vitro* formation of CL from linoleoyl-CoA and MLCL, and increased mitochondrial respiratory chain complex protein subunits. Expression of αTFP or MLCL AT-1 in control or BTHS lymphoblasts cultured in the presence of linoleate elevated L_4_-CL levels and other linoleate containing CL molecular species. Interestingly, expression of αTFP or MLCL AT-1 increased formation of non-linoleate tetra-acyl CL species as well. This suggests that, at least in lymphoblasts, αTFP and MLCL AT-1 may utilize a variety of MLCL and acyl-Coenzyme A species. The greater formation of overall CL mass in αTFP and MLCL AT-1 transfected BTHS lymphoblasts compared to control cells was likely due to the presence of higher levels of endogenous MLCL observed in BTHS cells which would most likely provide a larger pool of endogenous substrate [19]. Interestingly, RNAi knock down of αTFP in BTHS lymphoblasts resulted in a further accumulation of MLCL. This observation might partially explain why BTHS cells are not completely devoid of CL as αTFP could reacylate at least a portion of the accumulated MLCL. The above data suggest that elevation of αTFP in BTHS cells may serve as a potential therapeutic approach to treat BTHS.

The polyclonal antibody to the pig liver mitochondrial MLCL AT cross-reacted with both human MLCL AT-1 [22] and in the current study with human αTFP. It is likely that this antibody recognized identical epitopes on both MLCL AT-1 and αTFP. This was not surprising due to the observed sequence homology between αTFP and MLCL AT-1 as indicated in **Figure 1**. The observation that T_4_-treatment resulted in both an increase in MLCL AT-1 and αTFP protein expression in mitochondrial fractions prepared from rat liver suggested that MLCL AT-1 and αTFP may be related and that MLCL AT-1 could be derived from αTFP. Knock down of αTFP in HeLa cells using RNAi generated from upstream nucleotide regions 243–267 and 283–307 not present in MLCL AT-1 resulted in a decrease in expression of αTFP mRNA. However, this did not alter MLCL AT-1 mRNA expression. These results suggest that MLCL AT-1 is possibly a splice variant of the αTFP gene. It is tempting to speculate on why there are splice variants with MLCL-AT activity. It is possible that they may reside in different compartments within the mitochondrion, or have some preference for particular molecular species of MLCL.

Trifunctional protein was identified as a multifunctional, membrane-bound beta-oxidation enzyme protein catalyzing three enzyme activities - long-chain enoyl-Coenzyme A hydratase, long-chain 3-hydroxyacyl-Coenzyme A-dehydrogenase and long-chain 3-oxoacyl-Coenzyme A thiolase [1], [2], [34], [35]. Mitochondrial trifunctional protein deficiency is a rare autosomal recessive fatty acid oxidation disorder which may result in sudden infant death, a Reye-like syndrome, cardiomyopathy or skeletal muscle myopathy [36], [37]. However, fatty acid oxidation disorders may account for as much as approximately 5% of all cases of sudden infant death [38]. Cardiomyopathy is a major clinical finding of BTHS [20], [21]. In addition, a potential link between β-oxidation defects and BTHS has been proposed [39]. Similar to BTHS, early and correct diagnosis of fatty acid oxidation disorders allows for the appropriate early dietary and pharmacological intervention, which may have major effects on outcome [40]. However, a reduction in CL levels has not been observed in fatty acid oxidation disorders. Mutations in αTFP have been documented in trifunctional protein deficiency [41]. However, a reduction of L_4_-CL in alpha subunit mutations has never been demonstrated most likely since these patients have functional tafazzin.

Data from shotgun lipidomic analyses of mammalian tissue mitochondrial lipidomes, to model CL remodeling, predicted that both acyltransferase and transacylase activities as well as acyl selectivity’s play key roles in cardiolipin remodeling [42]. A recent study identified a novel mitochondrial protein, Them5, which exhibits thioesterase activity with long-chain acyl-CoAs and a strong substrate preference for C18 polyunsaturated fatty acids [43]. *Them5^−/−^* mice exhibit an increase in MLCL implicating thioesterase activity in the regulation of CL remodeling. Indeed, several acyltransferases and transacylases involved in mammalian cardiolipin remodeling have been identified including MLCL AT-1, ALCAT1 and tafazzin [22], [25], [44]–[46]. A recent study indicated that human recombinant TFP interacted strongly with CL and phosphatidylcholine suggesting that the natural TFP complex associates with the inner mitochondrial membrane through direct interactions with phospholipids [47]. In addition, TFP expression was suppressed by ALCAT1 overexpression in H9c2 cell lines and upregulated by ALCAT1 deficiency in liver of ALCAT1 null mice [48]. We hypothesize that the αTFP may contribute to a novel and distinct enzymatic activity in addition to the three activities already associated with trifunctional protein and thus links an enzyme of mitochondrial β-oxidation to CL remodeling. Similar to MLCL AT-1, αTFP does not contain conserved motifs observed in classical 1-acylglycerophosphate acyltransferases [49]. However, newly identified enzymatic activities for existing enzymes are continuing to emerge. For example, the recent discovery of the oxidase activity of mammalian catalase; an enzyme previous studied comprehensively for over 100 years [50]. In summary, we show that mitochondrial αTFP exhibits both *in vitro* and *in vivo* MLCL AT activity linking an enzyme of mitochondrial β-oxidation to CL remodeling.
